# Developing and predicting of early mortality after endovascular thrombectomy in patients with acute ischemic stroke

**DOI:** 10.3389/fnins.2022.1034472

**Published:** 2022-12-20

**Authors:** Yimin Chen, Sijie Zhou, Shuiquan Yang, Mohammad Mofatteh, Yuqian Hu, Hongquan Wei, Yuzheng Lai, Zhiyi Zeng, Yajie Yang, Junlin Yu, Juanmei Chen, Xi Sun, Wenlong Wei, Thanh N. Nguyen, José Fidel Baizabal-Carvallo, Xuxing Liao

**Affiliations:** ^1^Department of Neurology and Advanced National Stroke Center, Foshan Sanshui District People’s Hospital, Foshan, Guangdong, China; ^2^Department of Surgery of Cerebrovascular Diseases, The First People’s Hospital of Foshan, Foshan, Guangdong, China; ^3^School of Medicine, Dentistry and Biomedical Sciences, Queen’s University Belfast, Belfast, United Kingdom; ^4^The First School of Clinical Medicine, Southern Medical University, Guangzhou, Guangdong, China; ^5^Department of 120 Emergency Command Center, Foshan Sanshui District People’s Hospital, Foshan, Guangdong, China; ^6^Department of Neurology, Guangdong Provincial Hospital of Integrated Traditional Chinese and Western Medicine, Nanhai District Hospital of Traditional Chinese Medicine of Foshan City, Foshan, Guangdong, China; ^7^Department of Scientific Research and Education, Foshan Sanshui District People’s Hospital, Foshan, Guangdong, China; ^8^The First School of Clinical Medicine, Southern Medical University, Foshan, China; ^9^School of Laboratory Medicine and Biotechnology, Southern Medical University, Foshan, China; ^10^Second Clinical College, Guangzhou Medical University, Guangzhou, China; ^11^School of Medicine, Shaoguan University, Shaoguan, Guangdong, China; ^12^Medical Intern, Foshan Sanshui District People’s Hospital, Foshan, Guangdong, China; ^13^Department of Neurology, Radiology, Boston University Chobanian & Avedisian School of Medicine, Boston, MA, United States; ^14^Department of Neurology, Baylor College of Medicine, Parkinson’s Disease Center and Movement Disorders Clinic, Houston, TX, United States; ^15^Department of Sciences and Engineering, University of Guanajuato, León, Mexico; ^16^Department of Neurosurgery and Advanced National Stroke Center, Foshan Sanshui District People’s Hospital, Foshan, Guangdong, China

**Keywords:** ischemic stroke, endovascular therapy, thrombectomy, intracranial hemorrhage, mortality

## Abstract

**Background:**

Stroke is one of the leading causes of mortality across the world. However, there is a paucity of information regarding mortality rates and associated risk factors in patients with acute ischemic stroke (AIS) undergoing endovascular thrombectomy (EVT). In this study, we aimed to clarify these issues and analyzed previous publications related to mortality in patients treated with EVT.

**Methods:**

We analyzed the survival of 245 consecutive patients treated with mechanical thrombectomy for AIS for which mortality information was obtained. Early mortality was defined as death occurring during hospitalization after EVT or within 7 days following hospital discharge from the stroke event.

**Results:**

Early mortality occurred in 22.8% of cases in this cohort. Recanalization status (modified thrombolysis in cerebral infarction, mTICI) (*p* = 0.002), National Institute of Health Stroke Scale Score (NIHSS) score 24-h after EVT (*p* < 0.001) and symptomatic intracerebral hemorrhage (sICH) (*p* < 0.001) were independently associated with early mortality. Age, sex, cardiovascular risk factors, NIHSS score pre-treatment, Alberta Stroke Program Early CT Score (ASPECTS), stroke subtype, site of arterial occlusion and timing form onset to recanalization did not have an independent influence on survival. Non-survivors had a shorter hospitalization (*p* < 0.001) but higher costs related to their hospitalization and outpatient care.

**Conclusion:**

The recanalization status, NIHSS score 24-h after EVT and sICH were predictors of early mortality in AIS patients treated with EVT.

## Introduction

Stroke is one of the leading causes of death and disability worldwide. For this reason, low mortality and good functional status are important goals in clinical trials assessing the outcome of patients with acute ischemic stroke (AIS). Mortality in patients with stroke may have diverse causes, but evidence shows that it may be linked to early neurological complications such as recurrent stroke, hemorrhagic transformation or malignant edema, as well as systemic complications in the days following the acute event (Langhorne et al., 2000; Indredavik et al., 2008; Linfante et al., 2015; Rohweder et al., 2015; Viderman et al., 2020). A large study enrolling 13,721 European patients showed that 25.2% of cases experienced one or more medical complications during the hospitalization in the days following AIS (Ingeman et al., 2011; Rocco et al., 2012).

Stroke severity at onset and patient age have been estimated as some of the most important predictive variables for prognosis and post-stroke complications (Ingall, 2004; Indredavik et al., 2008). Moreover, occlusion site plays also a major role, as large vessel occlusion (LVO), i.e., internal carotid artery and/or proximal middle cerebral artery have a worse prognosis compared with more distal or medium vessel occlusions (Karamchandani et al., 2021). For example, a study including 764 patients with LVO showed a 90-day mortality rate of 26% (Karamchandani et al., 2021). Older age, higher National Institute of Health Stroke Scale Score (NIHSS) at presentation and greater modified Rankin Score (mRS) at discharge were predictors of mortality; however, patients undergoing revascularization therapy showed a survival benefit (Karamchandani et al., 2021). As large vessel recanalization is a modified variable, efforts have been done to achieve expedited and effective recanalization in patients with AIS and LVO (Broderick et al., 2013; Ciccone et al., 2013; Kidwell et al., 2013). Since 2014, randomized clinical trials demonstrated the benefit of endovascular thrombectomy (EVT) compared with standard therapy by using novel stent retrievers in functional outcome, assessed by the modified Ranking Scale (mRS) at 90 days (Goyal et al., 2016). Therefore, EVT is currently considered the standard of care for AIS with LVO by most health agencies in developed and developing countries. However, mortality rate at 90 days did not differ in a meta-analysis of 1,287 patients (634 EVT; 653 controls) including five randomized clinical trials: MR CLEAN, ESCAPE, REVASCAT, SWIFT PRIME, and EXTEND IA (Goyal et al., 2016; Eskey et al., 2018).

We aimed to investigate early mortality following EVT. We defined early mortality as inpatient mortality after EVT or death within 7 days after hospice home discharge as some local customs preferred patients pass away at home.

## Materials and methods

This retrospective study enrolled prospectively collected data of consecutive AIS patients with large vessel occlusion (LVO) who underwent EVT from October 2019 to February 2022 at two comprehensive stroke centers in China. Data were obtained from the Big Data Observatory Platform for Stroke in China and from the individual hospital data platforms. Inclusion criteria for this study were as follows: (1) EVT for patients with LVO including internal carotid artery (ICA), tandem, proximal middle cerebral artery (M1 and M2 segments), basilar artery, and other big vessels treated within 24 h of symptom onset; (2) age ≥ 18 years old. Patients with missing follow-up data were excluded. From 80 to 90% of patients underwent EVT using the SOLITAIRE system, the rest were treated with previous generation of devices, such as MERCI and PENUMBRA.

The following data were collected: age, sex, vascular risk factors [including hypertension, diabetes mellitus, coronary artery disease (CAD), atrial fibrillation (AF), prior stroke, chronic kidney disease (CKD), and dyslipidemia and smoking]. The NIHSS pre-treatment was calculated by stroke neurologists at each center. The Alberta Stroke Program Early CT Score (ASPECTS) before treatment was assessed on CT scans as reported by the site.

Door-to-needle time (DNT) and onset-to-needle time (ONT) were recorded for those receiving IV thrombolysis. Door-to-puncture time (DPT), door-to-recanalization time (DRT), puncture-to-recanalization time (PRT), and last known normal-to-puncture-time (LKNPT) were assessed for patients receiving EVT. Reperfusion was estimated with the modified thrombolysis in cerebral infarction (mTICI), accordingly as absent or minimal reperfusion: 0/1; partial arterial filling <50%: 2a; partial arterial filling: 2b; near complete reperfusion: 2c; and complete reperfusion: 3. Successful reperfusion was defined as mTICI post-EVT, 2b or 3. The NIHSS score was assessed 24-h following EVT. Symptomatic intracranial hemorrhage (sICH) was defined as intracranial hemorrhage associated with neurological deterioration post EVT manifested by an increase of ≥4 points in the NIHSS score (Rao et al., 2014).

### Outcome measures

The outcome variable was “early mortality” defined as death during hospitalization following EVT or death within 7 days following hospice home discharge. We consider this time important, as it provides an opportunity window to decrease death when patients are still under thorough medical care. Mortality corresponded to a mRS of six. Stroke neurologists and nurses followed patients by telephone calls or in-person consultations to assess the patient’s outcome.

### Ethics

The Institutional Review Board of both hospitals approved the study protocol. Written informed consent from the participants’ legal guardian/next of kin was obtained to perform the EVT.

### Statistical analysis

We summarized data in percentages, means and standard deviations for normally distributed and medians with interquartile range (IQR) for non-normally distributed variables. The chi-square (χ2) and Fisher’s exact tests were used to compare proportions between groups. The student’s *t*-test and Mann–Whitney *U*-test were used to compare normally and non-normally distributed continuous variables, respectively. Statistically significant variables (*p* < 0.05) in the bivariate analysis were included as independent variables in a multivariate logistic regression model using a Wald backward method to assess their effect on the dependent variable: Early mortality. Elimination threshold from the final model was a *p*-value ≥ 0.10. Unstandardized B coefficients and exponentiation of B (ExpB) coefficient with 95% confidence intervals (CI) were used to provide an estimated weight of the independent variable. Goodness of fit of the final regression model was evaluated with the Hosmer–Lemeshow test, *p*-value < 0.05 was considered poor fit. Determination coefficients (R2) were calculated for the final regression model. If the *P*-value was less than 0.05, results were considered statistically significant. IBM SPSS version 26 (IBM-Armonk, NY) was used for statistical analyses.

## Results

We enrolled 249 consecutive patients with AIS who underwent EVT. Four patients were excluded from the final analysis owing to loss to follow-up. A total of 245 patients were analyzed, among them, 56 patients died during the study period (22.8%). Patients who died had a median length (IQR) of hospitalization of 4 (2, 6) days.

Patients who died were on average 4.3 years older than those who survived (*p* = 0.029) and had a median of 5 points higher NIHSS score pre-treatment on admission (*p* < 0.001). This difference in NIHSS widened to 14 points, 24-h following EVT (*p* < 0.001) (Table 1). Patients who survived, achieved a much higher percent of optimal recanalization (mTICI ≥ 2b) compared to those who died: 91.53 vs. 60.7% (*P* < 0.001). The timing between symptom onset and initiation of reperfusion did not differ either, except for a shorter PRT (*p* = 0.013) in survivors. A total of 24 patients from the cohort suffered symptomatic intracerebral hemorrhage (sICH) (9.79%). The frequency of sICH was markedly higher in patients who did not survive 33.9 vs. 2.65% (*p* < 0.001). Length of hospitalization was shorter in patients who died as expected; however, costs of hospitalizations were higher in patients who died during the study period (Table 1). Other variables such as sex, cardiovascular risk factors, site of arterial occlusion, stroke subtypes and complicated lung infection did not differ between survivors and non-survivors.

**TABLE 1:** Summary of demographic, clinical hemodynamic, and therapeutic features between non-surviving and surviving patients.

	Early mortality	Survival	X^2^/t/z	*P*-value
Number	56	189		
Age (years), mean ± SD	67.82 ± 11.94	63.52 ± 13.09	2.200	0.029
Female, *n*%	17 (30.36)	57 (30.16)	0.001	0.977
Hypertension, *n*%	34 (60.71)	108 (57.14)	0.226	0.634
DM, *n*%	15 (26.79)	41 (21.69)	0.635	0.425
CAD, *n*%	12 (21.43)	29 (15.34)	1.148	0.284
AF, *n*%	21 (37.5)	64 (33.86)	0.252	0.615
Prior stroke, *n*%	13 (23.21)	36 (19.05)	0.469	0.494
CKD, *n*%	7 (12.50)	13 (6.88)	1.821	0.177
Dyslipidemia, *n*%	7 (12.50)	30 (15.87)	0.383	0.535
Smoker, *n*%	9 (16.07)	37 (19.58)	0.348	0.555
NIHSS pre-treatment (IQR)	20.00 (16.00, 22.00)	15.00 (11.00, 18.00)	5.416	<0.001
ASPECTS pre-treatment (IQR)	8.00 (8.00, 9.00)	9.00 (8.00, 9.00)	1.286	0.199
IV thrombolysis, *n*%	26 (46.43)	71 (37.57)	1.419	0.234
DNT (IQR)	52.00 (32.75, 59.75)	42.00 (31.50, 56.75)	1.487	0.137
ONT (IQR)	140.00 (89.00, 200.00)	135.00 (101.75, 178.75)	0.209	0.835
**TOAST classification**				
Large artery atherosclerosis	25 (44.6)	105 (55.6)	2.221	0.528
Cardioembolic	29 (51.8)	77 (40.7)		
Stroke other determined cause	1 (1.8)	3 (1.6)		
Undetermined cause	1 (1.8)	4 (2.1)		
**Site of arterial occlusion**				
Distal/terminal ICA, *n*%	14 (25.00)	30 (15.87)	8.785	0.118
MCA-M1, *n*%	14 (25.00)	87 (46.03)		
MCA-M2, *n*%	4 (7.14)	11 (5.82)		
Tandem, *n*%	9 (16.07)	27 (14.29)		
Basilar, *n*%	12 (21.43)	29 (15.34)		
Others, *n*%	3 (5.36)	5 (2.56)		
ICA and MCA-M1, *n*%	37 (84.09)	144 (76.19)	2.192	0.130
DPT (IQR), minute	145.00 (114.25, 196.00)	148.00 (116.00, 196.00)	0.151	0.880
DRT (IQR), minute	247.00 (171.25, 322.00)	231.00 (173.50, 285.00)	1.061	0.289
PRT (IQR), minute	85.00 (44.75, 121.50)	60.00 (40.00, 92.50)	2.492	0.006
LKNPT (IQR), minute	302.50 (208.75, 493.75)	310.00 (212.50, 440.50)	0.202	0.840
mTICI post-EVT ≥ 2b, *n*%	34 (60.71)	173 (91.53)	31.313	<0.001
NIHSS 24-h post-EVT	25.00 (21.25, 28.00)	11.00 (5.00, 15.00)	8.873	<0.001
Pulmonary infection, *n*%	28 (50.00)	79 (41.80)	1.181	0.277
sICH, *n*%	19 (33.93)	5 (2.65)	47.844	<0.001
Length of hospitalization (days) (IQR)	4.00 (2.00, 6.00)	14.00 (10.00, 23.00)	7.566	<0.001
Hospitalization costs (RMB) (IQR)	117455.52 (83506.09, 164035.68)	112.389.72 (87487.21, 153096.45)	0.414	0.679

AF, atrial fibrillation; ASPECTS, Alberta stroke program early CT score; CAD, coronary artery disease; CKD, chronic kidney disease; DM, diabetes mellitus; DPT, door-to-puncture time; DRT, door-to-recanalization time; EVT: endovascular thrombectomy; LKNPT, last-known normal-to-puncture time; mTICI, modified thrombolysis in cerebral infarction; NIHSS, National Institute of Health Stroke Scale/score; PRT, puncture-to-recanalization time; sICH, symptomatic Intracranial Hemorrhage; TOAST, trial of ORG 10,172 in acute stroke treatment.

In the multivariate logistic regression NIHSS score 24-h after EVT (*p* < 0.001), mTICI post-EVT ≥ 2b (*p* = 0.002); and sICH (*p* < 0.001) were significantly associated with early mortality (Table 2). Age, pre-treatment NIHSS score and PRT were excluded from the final regression model. Length and costs of hospitalization were not considered in the regression model as these variables depend on patient survival.

**TABLE 2:** Logistic regression analysis of statistically significant variables in the bivariate analysis.

Variables in the final equation	B	Exp^B^ coefficient	Exp^B^ 95% C.I.	Wald	*P*-value
NIHSS 24-h post-EVT	0.188	1.207	1.137–1.282	38.096	<0.001
mTICI post-EVT ≥ 2b	–1.556	0.211	0.080–0.559	9.801	0.002
sICH	2.308	10.05	2.965–34.062	13.728	<0.001

Constant: B = −3.802, Exp^B^: 0.22, p = 0.000. Hosmer–Lemeshow: *X*^2^ = 5.343 (*p* = 0.618). Variables excluded in the final model: age (p = 0.297) pre-treatment NIHSS score (p = 0.941), and puncture-to-recanalization time (PRT) (p = 0.556). Cox and Snell *R*^2^ = 0.392; Nagelkerke *R*^2^ = 0.595.

## Discussion

**FIGURE 1:**
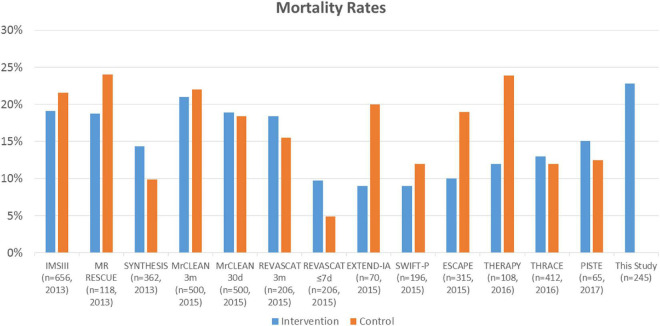
Summary of randomized clinical trials assessing the effect of endovascular thrombectomy (EVT) compared with standard care on mortality rates and this study. Blue: intervention and EVT. Orange: controls and standard therapy.

In the present study, early mortality was noted in 22.8% of patients who had undergone EVT for acute ischemic stroke. High mortality rates among patients treated with EVT is mostly observed in patients with LVO (Malhotra et al., 2017). These patients represent about one third of cases, but account for three fifths of post-stroke dependence and death (Malhotra et al., 2017). Compared with non-LVO patients, those with LVO had an estimated mortality rate of 26.2 vs. 1.3% (OR: 4.1; 95% CI: 2.5–6.6, *p* < 0.0001) (Malhotra et al., 2017). In a meta-analysis of randomized clinical trials of EVT vs. medical care alone, there was no difference in the mortality proportion between groups: 248/1,538 (16.12%) vs. 234/1,342 (17.43%), *p* = 0.52 (Figure 1; Rodrigues et al., 2016). These proportions were similar for trials published in 2013 where early generation devices were used for thrombectomy such as the Merci retriever, Penumbra system or aspiration systems. For trials published in 2015 and later, including MR CLEAN; REVASCAT, EXTEND-IA, SWIFT PRIME, ESCAPE, THRACE, and PISTE, modern stent retrievers were used in more than 86% of cases (Berkhemer et al., 2015; Campbell et al., 2015; Goyal et al., 2015; Jovin et al., 2015; Saver et al., 2015; Bracard et al., 2016; Mocco et al., 2016; Muir et al., 2017). The latter suggest that use of early vs. latter generation retrievers do not have a major influence on mortality.

Of note, outcomes of clinical trials of patients undergoing mechanical thrombectomy may differ from real life experience (Zaidat et al., 2018; Qureshi et al., 2020). A study enrolling 23,375 patients treated with mechanical thrombectomy, showed lower in-hospital mortality rates for those participating in a clinical trial (*n* = 430, 1.8%): OR 0.14; 95% CI: 0.03–0.71, *p* < 0.001 (Qureshi et al., 2020). This difference is key when generalizing results from clinical trials and observational studies to real life experience.

An important question is which variables can predict mortality in patients undergoing EVT. In our study, older age, greater clinical severity at presentation and at 24 h (NIHSS score), higher PRT, lower mTICI, and presence of sICH were observed in early mortality group patients. However, in the multivariate regression only higher NIHSS score at 24 h, lower mTICI and sICH were independently associated with early mortality. It is likely, however, that the NIHSS score at 24 h may depend on the degree of revascularization or early occurrence of sICH. A study of 111 patients with anterior and posterior AIS treated with EVT from 2012 to 2017, reported a mortality rate of 37.8% within 3 months following the procedure. Non-surviving patients had higher NIHSS scores at presentation, compared with survivors: 19.6 vs. 14.9 (*p* < 0.002), lower rates of revascularization: 50 vs. 78.3%, higher rates of hemorrhagic conversion: 47.6 vs. 21.7%, technical problems during the procedure: 26.2 vs. 7.4% and higher rates of cancer history (Awad et al., 2020). This study mirrors our results related to revascularization status and frequency of sICH. While we did not assess for technical problems, a longer PRT may reflect difficulties to achieve rapid recanalization in our study. A multidisciplinary well-coordinated team is helpful to shorten PRT (Chen et al., 2022b; Yang et al., 2022). An additional study also showed that higher NIHSS scores at baseline and lower rates of recanalization following EVT were consistently associated with inpatient mortality (Mouchtouris et al., 2019). Whereas when assessing mortality following EVT 6–16 h after last normal in the DEFUSE 3 (The Endovascular Therapy Following Imaging Evaluation for Ischemic Stroke 3) trial, age older than 75 years, NIHSS score of ≥20, wake-up stroke and diabetes predicted death within 90 days (Taussky et al., 2021). While the NIHSS score on arrival is important for prognosis in studies not undergoing an active recanalization procedure, it is likely that the 24-h NIHSS score has a greater weight as it reflects irreversible damage and, in some cases, life-threatening conditions such as sICH, severe edema, brain herniation, etc. We did not find ASPECTS as a predictor of mortality neither.

**FIGURE 2:**
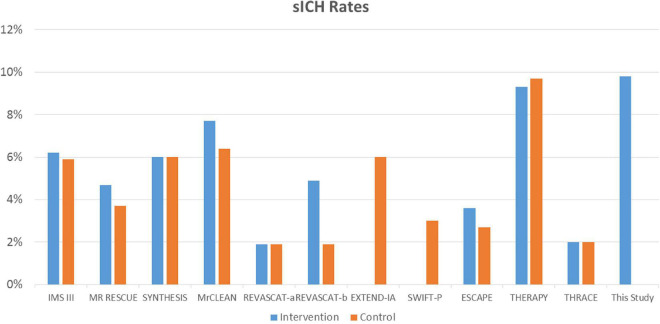
Summary of randomized clinical trials assessing the effect of endovascular thrombectomy compared with standard care on sICH rates and this study. Blue: intervention and EVT. Orange: controls and standard therapy. REVASCAT-a: SITS-MOST criteria; REVASCAT-b: ECASS II criteria. PISTE study: 0% sICH, not included in the figure.

In our study, sICH was strongly associated with mortality; however, this has not been observed in all studies. For example, one retrospective study did not find an association between inpatient mortality and hemorrhagic conversion with significant mass effect (Mouchtouris et al., 2019). However, another study including 417 patients who had undergone successful recanalization showed a significant association between sICH and mortality (OR 9.51; 95% CI: 4.54–19.92). This variable had much greater weight on mortality at 3-months compared with age, poor pre-treatment collateral status, baseline blood glucose and the NIHSS score, which were also significant (Zhang et al., 2020). This is in accordance with the findings of our study, where sICH had the higher weight to predict early mortality. Moreover, sICH was more frequently present in our study compared with randomized clinical trials of EVT in AIS, with rates similar to those observed in the THERAPY trial, which enrolled 108 patients; 55 underwent EVT and 53 controls (Figure 2; Mocco et al., 2016). Increased peak body temperature (≥38°C) during the acute phase has been associated with increased risk of sICH and poorer outcome in stroke patients with large artery disease undergoing EVT (Chen et al., 2022a).

In our study, we did not find a statistically significant difference in the rate of pulmonary infections in non-survivors compared with survivors. Pneumonia has been deemed as the most common cause of mortality up-to 1 year after AIS (Ingeman et al., 2011; Rocco et al., 2012). This data suggests that systemic complications may explain most of late mortality (i.e., after 3 months) in patients with AIS when a large proportion of patients have been already discharged from the hospital. Although non-survivors in our study had a shorter hospitalization time than survivors (*p* < 0.001), costs of hospitalization were higher for non-survivors.

Other variables may also influence the mortality rate in patients treated with EVT. For example, experience-performing EVT is another variable potentially influencing the outcome. In a Nationwide analysis in the US, it was estimated that for every 10 more proceduralist cases, patients had 4% lower inpatient mortality (*p* < 0.0001), and 3% greater odds of good outcome (Stein et al., 2021). In contrast, whether the risk of sICH has a link with the device used for thrombectomy or the technical experience of the treating physician should be further clarified. Moreover, patients undergoing AIS show a remarkable increase in serum inflammatory markers that may potentially increase the risk of death. Evidence has shown that high dose atorvastatin may decrease such markers and improve functional outcome (Tuttolomondo et al., 2016). The latter finding suggests that inflammation may have an important role in mortality in AIS. However, other markers of inflammation such an arterial stiffness may also be explored in relation with early mortality in this scenario (Tuttolomondo et al., 2017; Zanoli et al., 2017).

Our study has limitations. It is a two-center study with a limited sample size. Further large-scale, multi-center investigations with larger sample sizes are required to verify these findings. Our study included patients of Asian origin and it is unclear whether these patients represent a population with a high risk of death after EVT. A recent study, for example, showed higher mortality rates in Blacks compared to White patients treated with EVT (Catapano et al., 2021). Another limitation is that we did not record the exact day an individual patient died and the cause of death on each case, these variables could be addressed in further studies. Despite these limitations, we were able to identify the factors associated with early mortality after EVT.

## Conclusion

Early mortality occurred in 22.8% of the real-world cohort of patients treated with EVT. Poor recanalization following EVT, poor clinical recovery or deterioration at 24-h and sICH seem to have a major influence in this outcome. Although patients suffering early mortality had shorter hospitalization times, it may represent higher costs and a greater burden for health care systems.

## Data availability statement

The original contributions presented in this study are included in the article/supplementary material, further inquiries can be directed to the corresponding authors.

## Ethics statement

The studies involving human participants were reviewed and approved by The First People’s Hospital of Foshan Review Board and Foshan Sanshui District People’s Hospital Review Board. The patients/participants provided their written informed consent to participate in this study.

## Author contributions

YC, JC, and XL: conception and design and drafting the manuscript. YC, SZ, SY, YH, HW, YL, ZZ, YY, JY, JC, XS, and WW: acquisition of data. MM and TNN: review and critique. JB-C: conception, drafting of the manuscript, review, and critique. All authors contributed to the article and approved the submitted version.
